# Prevalence of physical activity involvement among older adults in Africa: A scoping review

**DOI:** 10.4102/hsag.v31i0.3378

**Published:** 2026-09-25

**Authors:** Ernest A. Bekoe, Tawagidu Mohammed, Bashir Bello, Nontembiso Magida

**Affiliations:** 1 Department of Physiotherapy, Faculty of Healthcare Sciences, University of Pretoria, Pretoria, South Africa; 2 Department of Physiotherapy, College of Health, University of Ghana, Accra, Ghana; 3 Department of Physiotherapy, Faculty of Health Sciences, Bayero University, Kano, Nigeria; 4 Lifestyle Disease Research Entity, Faculty of Health Sciences, North-West University, Mafikeng Campus, South Africa

**Keywords:** barriers, facilitators, healthy ageing, involvement, older adults, physical activity, prevalence

## Abstract

**Background:**

The global rise in the population of older adults has significant health implications, particularly in Africa, where demographic transitions are accelerating. Reduced participation in physical activity (PA) is a key determinant of chronic disease burden, functional decline and poor quality of life. Despite its importance, evidence on PA participation among older adults in Africa remains fragmented.

**Aim:**

To synthesise evidence on the prevalence of PA involvement among older adults in Africa.

**Methods:**

Peer-reviewed studies were systematically searched across PubMed, SCOPUS, Web of Science, CINAHL and Google Scholar. Search terms combined prevalence, older adults, physical activity and participation. Eligible studies included studies of older adult populations that reported PA prevalence. Three reviewers independently screened and appraised the studies. Data were charted and synthesised narratively.

**Results:**

Seven studies revealed significant variability in physical activity levels among older adults in Africa, with low engagement in South Africa and Ethiopia, while Ghana showed higher levels of vigorous activity. The main barriers included advanced age, low income, obesity and a lack of awareness, whereas encouragement from family and healthcare professionals helped enhance participation.

**Conclusion:**

In general, physical activity levels among older adults remain low and are influenced by contextual factors, underlining the importance of addressing social and economic challenges.

**Contribution:**

This review contributes by consolidating evidence on physical activity among older adults in Africa, exposing research gaps and practical needs. Based on these insights, healthcare providers should encourage activity, give clear advice and work with communities to strengthen support systems.

## Introduction

Older adults are at an increased risk of non-communicable diseases and are often plagued with diminishing functional reserves as well as age-associated conditions such as dementia and loneliness (Lindsay-Smith et al. 2019; Van Dijk-Huisman et al. 2022; Zhou et al. 2024,). The increased burden of this age-associated effect on older adults presents challenges to social and healthcare structures, which invariably affect longevity and quality of life (Lindsay-Smith et al. 2019). The World Health Organization (WHO) explains physical activity (PA) as any bodily movement produced by skeletal muscles that require energy expenditure (Bull et al. 2020). In older adults, non-performance of PA predisposes to the adverse effects of ageing and functional decline, such as a decrease in cardiopulmonary fitness (Distefano & Goodpaster 2018).

Older adults are encouraged to engage in exercise-based physical activity including aerobic exercise, muscle strengthening, bone-strengthening activities, balance training and multi-component programmes (Piercy et al. 2018). Aerobic exercise has been shown to lower blood pressure, reduce modifiable risk factors for conditions such as diabetes and hypertension and improve cognitive function (Alpsoy 2020; Huang, Chen & Li 2023; Liu et al. 2019). Balance training is particularly important, as it reduces the risk of falls in older adults and enhances both reactive and proactive balance (Lesinski et al. 2015; Sherrington et al. 2019). Strength training should target both the upper body and lower body, using resistance bands, free weights or bodyweight exercises to build functional capacity (Sandercock, Moran & Cohen 2022).

Although older adults are encouraged to perform PA to reduce the risk of chronic diseases and slow metabolic and muscle decline, their participation rates remain low and steadily decline with advancing age (Cunningham et al. 2020; Distefano & Goodpaster 2018; Kyu et al. 2016; Shur et al. 2021; You et al. 2021). For example, a meta-analysis conducted in Africa indicated a 45% prevalence of physical inactivity among adults, noting that being aged 60 years and above was a significant physical inactivity predisposing factor (odds ratio [OR] = 2.18, 95% confidence interval [CI]: 1.98–4.77) (Belete, Yirsaw & Taye 2026). Rates of decline in PA and poor adherence to standard recommendations among older adults in Ghana are evident; for instance, 61.1% did not meet WHO guidelines, and only 30.3% engaged in vigorous activity (Awlime-Ableh et al. 2025). Similarly, PA participation among older adults in Nigeria was low, with only 21.8% meeting WHO recommendations, and participation declined significantly with increasing age (Faronbi et al. 2024). Indeed, this has been the trend across most countries globally (Cunningham et al. 2020; DiPietro et al. 2020; WHO 2021; You et al. 2021). It is projected that the proportion of older adults globally will increase from 1.4 billion in 2019 to 2.1 billion in 2050, with 1.7 billion of these older adults residing in developing countries (Bull et al. 2020). With this increasingly ageing population worldwide, it is worrisome that the majority of older adults participate less in PA.

Even though WHO guidelines on PA participation for older adults recommend that PA be done regularly and integrated into daily educational, occupational, community or home settings (Bull et al. 2020), as echoed in the United States (Lee, Jackson & Richardson 2017), performing some form of PA is better than being sedentary. Additionally, the author further expressed that PA should be performed at a higher frequency, with greater intensity or for a longer duration to obtain better health benefits (Lee et al. 2017). However, another author in the United Kingdom opined that a broader perspective on the definition of PA should be adopted rather than focusing solely on PA as ‘exercise’, which in turn can demotivate older adults (Ioannou et al. 2023). Sadly, guidelines for PA participation among older adults may not be readily available or adequately disseminated to the public, policy-makers and health practitioners (Balis et al. 2021).

The prevalence of PA participation among older adults has been examined in a variety of international contexts (Sun, Norman & While 2013). However, evidence specific to the African setting remains limited. To address this gap, the present scoping review sought to explore and synthesise available data on PA participation among older adults in Africa.

## Methods

This scoping review’s methodology adopted the five-stage model developed by Arksey and O’Malley (2005).

This framework has been further explained by Levac, Colquhoun and O’Brien (2010) to include quality appraisals and has been detailed in accordance with the aim of this study. A Preferred Reporting Items for Systematic Reviews and Meta-analysis extension for a scoping review (PRISMA-ScR) checklist was applied to include all relevant sections. A protocol for this review was prepared prospectively.

### Stage 1: Identifying the research question

The primary research question guiding this study was: ‘What is the prevalence of PA participation among older adults in Africa?’ Subsequent sub-questions guiding this review were: (1) *Does gender play a role in determining physical activity participation among older adults?* (2) *What are the attributes of older adults who partake in PA?* We adopted the Participant, Concept and Context (PCC) framework to determine the eligibility of the research question (Hong et al. 2018).

### Stage 2: Identifying relevant studies

An extensive search criterion was developed for this review, with the assistance of a librarian, to gather relevant studies. We searched electronic databases, including PubMed, Scopus, Web of Science, Cumulative Index to Nursing and Allied Health Literature (CINAHL) and Google Scholar, from 29 November 2024 to 14 January 2025. Keywords used were separated by Boolean terms ‘AND’, ‘OR’ and ‘NOT’. The final step was to search the reference lists of the identified articles. The initial list of keywords included ‘prevalence’, ‘older adults’, ‘physical activity’ and ‘participation’. We included studies published in English from 2014 to January 2025. Quantitative, qualitative and mixed-methods peer-reviewed articles published in English with a focus on the prevalence of PA participation among older adults in Africa or among Africans living within or outside Africa were included. Studies that did not provide specific data on the prevalence of PA among older adults were excluded. All studies meeting the criteria were uploaded to EndNote referencing software, and duplicates were removed.

### Stage 3: Study selection

Two researchers (EAB and BB) independently retrieved studies for inclusion and exclusion, as recommended by Levac et al. (2010). At the beginning, middle and end of the abstract review process, researchers convened to discuss any challenges or uncertainties related to the selection of studies and to make any necessary revisions to the search strategy. Three independent researchers (NM, TM and EAB) screened titles, abstracts and full texts. Discrepancies were resolved by consensus or referred to another reviewer (BB) for validation.

### Stage 4: Charting the data

A data extraction tool was designed to organise and sort all data retrieved from the articles during the scoping review process. Two independent reviewers, using the inclusion criteria as the basis, evaluated the data extraction tool. The relevant information extracted from the data included study characteristics (author[s], year of publication, study design, period, setting and sample size), as well as participant characteristics (age range, mean age, sex distribution, socioeconomic status, health status and educational level). Additionally, PA measurement, outcome data of PA prevalence, statistical analysis and key findings were also recorded. The first author (EAB) completed initial data charting, followed by a discussion among the additional three authors (NM, TM and BB) to reach a consensus on codes and major themes that emerged. The two authors (EAB and NM) then refined themes iteratively and clustered them into categories. The themes that emerged were then confirmed by additional authors (TM and BB) for transparency. This information was continuously updated throughout the course of this scoping review.

### Stage 5: Collating, summarising and reporting the results

Preferred Reporting Items for Systematic Reviews and Meta-analyses extension for a scoping review (PRISMA-ScR) guidelines were employed to report the screening results. This review adopted a qualitative analysis of the results of selected studies. Thematic analysis was used to analyse the qualitative data from the reviewed papers, synthesising and interpreting the critical themes and issues that arose from the selected studies. Data were extracted and presented in Figure 1 to align with the study’s objective.

**FIGURE 1:**
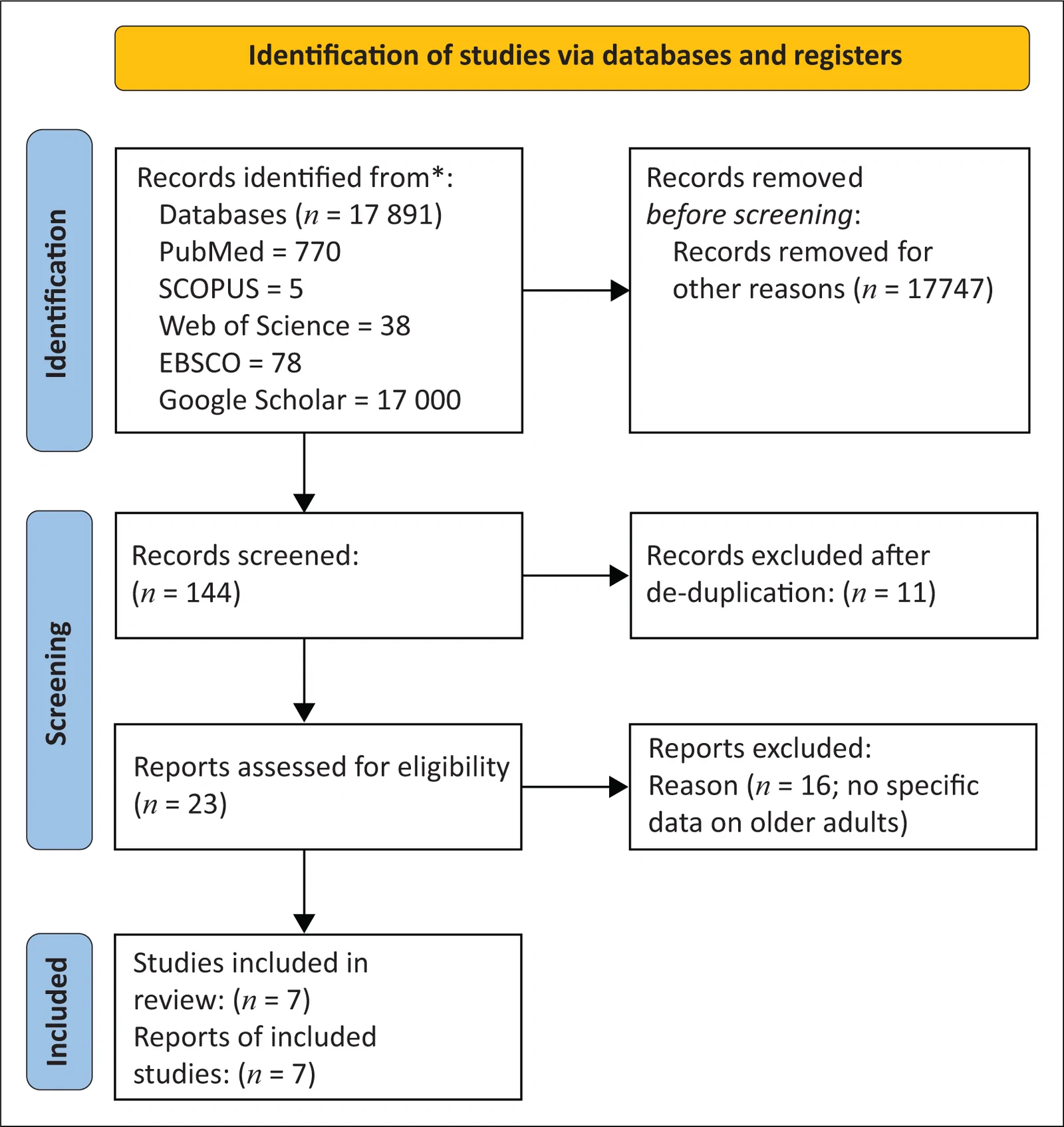
Flow diagram of Preferred Reporting Items for Systematic Reviews and Meta-analyses extension for a scoping review.

### Ethical considerations

Ethical clearance to conduct this study was obtained from the University of Pretoria Faculty of Health Sciences Research Ethics Committee on 09 July 2025. The ethical approval number is 397/2024.

## Review findings

The search conducted returned 17 891 records from the databases. The flowchart demonstrates that 144 articles were selected from the various databases for potential records to be screened for analysis. After removal of duplication, 133 articles were identified for screening by title and abstract, and then 23 articles were selected for full-text screening based on reported data on PA prevalence in African adults. Seven articles were selected for the final review as presented in Figure 1. These seven specifically reported on the prevalence of physical activity among older adults, as demonstrated in Figure 1.

### Study characteristics

This scoping review identified seven studies that reported on the prevalence of PA participation among older adults in Africa. The studies varied in their populations, methodologies and outcome measures, but together they provide a snapshot of how older adults across the continent engage in PA, the barriers they face and the implications for health outcomes (Table 1). Another study examined older adults aged 65 years and above across six low- and middle-income countries, including Ghana and South Africa, and found that nearly half (44.9%) did not achieve the WHO-recommended 150 min of moderate-to-vigorous physical activity per week (Vancampfort et al. 2018). Low PA participation was associated with a range of health conditions, such as mild cognitive impairment, chronic pain, stroke and poor self-rated health, underscoring the need for interventions that address both medical and social determinants of activity.

**TABLE 1:** Results of included studies in scoping review.

Authors (year)	Demographic information (age group, gender and socioeconomic status)	Study characteristics (design, sample size and setting)	Types of PA measured	Prevalence of PA	Barriers to PA	Facilitators to PA	Health outcomes associated with PA levels	Recommendations
Vancampfort et al. (2018)	≥ 65 years and above; mean age 73.3 (±11.4); 62.4% females; socio-economic status by country-specific wealth quantiles	Cross-sectional; *N* = 1455; community-dwelling adults in China, Ghana, India, Mexico, Russia, South Africa.	Moderate-to-vigorous PA (Global Physical Activity Questionnaire)	44.9% did not meet the WHO-recommended ≥ 150 min per week; no country-specific rates	Older age, MCI, pain, chronic obstructive pulmonary disease, stroke, slow gait, weak grip, poor health, disability, low social cohesion, male sex	Not reported.	Low PA linked with mild cognitive impairment, pain, chronic obstructive pulmonary disease, stroke, slow gait, weak grip, poor health	Promote social cohesion; address comorbidities, pain, strength, gait
Adeloye et al. (2022)	≥ 15 years; mean age from 22–53; Gender and socio-economic status were not reported	Population-based; *n* = 3814 (15 studies), Nigeria	Not specified	52.0% physically inactive; women 55.8%; urban dwellers 56.8%; prevalence increased with age groups (60–79 years)	Traders in Urban markets in Lagos State, Civil servants in Abakaliki, Ebonyi State, and middle-class professionals in Asaba, Delta State.	Rural dwellers in Egbeda, Oyo State	Not reported	Improve occupational policies, town planning, awareness and sociocultural considerations
Banson et al. (2023)	≥ 20 years; 62.3% females	Cross-sectional; *N* = 106; diabetes clinic, Ghana	Moderate-to-vigorous PA (IPAQ short)	Low PA 44%, moderate PA 44%, high PA 12%; sex-specific: *F* = 23.8% low, M = 20.2% low	Female sex	Not reported.	73.6% abdominal obesity. All had diabetes	Combine PA education with exercise therapy; use wearable PA trackers
Jacob et al. (2023)	≥ 65 years; mean age 72.6 (±11.5); 55.0% females; socio-economic status by income-based wealth quantiles	Cross-sectional; *N* = 14 585; community-dwelling adults in China, Ghana, India, Mexico, Russia, South Africa	Moderate-to-vigorous PA (Global physical activity questionnaire: leisure, occupational, transport domains)	8.9% met ≥ 150 min per week of leisure time physical activity; Ghana 9.0%, South Africa 5.2%	Older age, female sex, low wealth or education, underweight, obesity, activities of daily living, less occupational PA and active travel	Not reported	Leisure-time physical activity is negatively associated with sarcopenia	Promote strength or resistance training, recreational PA, culturally adapted health promotion
Chikafu and Chimbari (2020)	≥ 18 years; mean age 42.7 (± 17.4); 67% female; Females ≥ 60 = 20.5%	Cross-sectional; *N* = 392 (72 aged ≥ 60); isiZulu adults, South Africa	Moderate-to-vigorous PA (STEP-wise)	18.1% sufficient PA; 54.2% high PA; 27.8% low PA	Female sex, older age, obesity, underweight, low awareness, education, unemployment, marital status, and cohabitation	Being unmarried, employed, and advised to be active	Low PA in females ≥ 60	Promote PA as a primary health intervention; develop multilevel interventions in rural settings
Shiferaw et al. (2024)	≥ 60 years; 75.5% male	Cross-sectional; *N* = 838; Amhara community, Ethiopia	Moderate-to-vigorous PA (Global physical activity questionnaire, Metabolic equivalent-min per week)	65.6% < 600 MET- min per week; inactivity higher in females (32%) and ≥ 80 years (16%)	Female sex, older age, unemployment, low education, depression	Not reported	Depression linked with inactivity	Promote PA benefits among women; address depression in older adults
Asiamah et al. (2023b)	≥ 60 years; 50% female	Cross-sectional; *n* = 686; community-dwelling older adults, Ghana	Vigorous PA (Global Physical Activity Questionnaire II).	74% engaged in ≥ 30 min per day vigorous physical activity	Number of siblings, dependents, social ties	Large families and strong social networks	Participants engaging in ≥ 30 min per day vigorous physical activity did not report better health	Use health promotion programmes to improve knowledge

Note: Please see full reference list of this article: Bekoe, E.A., Mohammed, T., Bello, B. & Magida, N., 2026, ‘Prevalence of physical activity involvement among older adults in Africa: A scoping review’, *Health SA Gesondheid* 31(0), a3378. https://doi.org/10.4102/hsag.v31i0.3378 for more information.

PA, physical activity; WHO, World Health Organization; MET, metabolic equivalent of task; MCI, mild cognitive impairment; IPAQ, The International Physical Activity Questionnaire.

In Nigeria, a study pooled data from 15 population-based studies and reported that more than half of adults aged 15 years and older were physically inactive (52.0%) (Adeloye et al. 2022). Women (55.8%) and urban residents (56.8%) had higher inactivity rates compared with men and rural dwellers. Although this study aggregated age groups, the trend suggested an increase in inactivity with advancing age, highlighting a growing public health concern as Nigeria’s population ages.

Accordingly, one study of adults with diabetes in Ghana found that PA participation was particularly low in this group, with 44% classified as inactive and only 12% achieving high PA levels (Banson et al. 2023).

Women were more likely than men to report low activity, and the high prevalence of abdominal obesity among participants further reflected the compounding health risks of inactivity. The authors recommended incorporating PA education and exercise therapy into diabetes management.

Similarly, another study assessed older adults in six countries and reported that only 8.9% engaged in sufficient leisure-time PA, with particularly low levels observed in Ghana (9%) and South Africa (5.2%) (Jacob et al. 2023). The findings also revealed that older age, female sex, low wealth and difficulties with activities of daily living were significant barriers to participation. Importantly, low leisure-time PA was negatively associated with sarcopenia, emphasising the protective role of activity in maintaining muscle mass and function. A summary of the study characteristics is detailed in Table 1.

Similarly, another author, working in a rural isiZulu community in South Africa, found mixed levels of activity among adults, with 18.1% reporting sufficient PA, 54.2% reporting high levels and 27.8% reporting low levels (Chikafu & Chimbari 2020). Older age, female sex and unemployment were associated with inactivity, whereas being advised to be active was linked to higher activity levels. The author emphasised the importance of culturally tailored community-based interventions in rural African settings. In Ethiopia, a study observed that 65.6% of older adults achieved less than 600 metabolic equivalent of task (MET)-min of PA per week, classifying them as inactive (Shiferaw et al. 2024). Female sex, older age, low education and depression were significant predictors of inactivity. Moreover, depression was strongly associated with low PA participation, underscoring the bidirectional relationship between mental health and activity.

Finally, one study found that 74% of older Ghanaian adults engaged in vigorous PA for at least 30 min per day (Asiamah et al. 2023b). Social factors, such as having large families and strong support networks, were important facilitators. Interestingly, participants who engaged in vigorous PA did not necessarily report better health outcomes, suggesting that the type and context of activity may matter as much as frequency and intensity.

Across these studies, common barriers to PA participation include older age, female sex, chronic illness, functional limitations and lower socioeconomic status (Jacob et al. 2023; Shiferaw et al. 2024; Vancampfort et al. 2018). Facilitators included social support, health advice and strong community networks (Asiamah et al. 2023b; Chikafu & Chimbari 2020). The evidence highlights the complexity of participation in PA among older adults in Africa, influenced by biomedical, social and cultural factors. Importantly, while some populations demonstrated relatively high engagement in vigorous activity, large proportions remained physically inactive, signalling an urgent need for targeted interventions.

## Discussion

This scoping review synthesises evidence regarding the prevalence and, subsequent, barriers, facilitators and health outcomes of PA participation among older adults in Africa. The findings indicate that a significant proportion of older adults are physically inactive, with participation levels differing by country, population and context. These results are consistent with global concerns about inactivity in ageing populations and highlight the need for targeted interventions in Africa. The studies reviewed consistently highlight low PA participation among older African adults. A study on low- and middle-income countries reported that nearly half of older adults across six countries, including Ghana and South Africa, failed to meet WHO recommendations for PA participation (Vancampfort et al. 2018). In addition, one study found even lower engagement in leisure-time PA participation, with only 8.9% meeting guidelines and particularly low prevalence in South Africa (5.2%) (Jacob et al. 2023). Similarly, another study observed high levels of inactivity in Ethiopia, where almost two-thirds of older adults fell short of 600 MET-min per week (Shiferaw et al. 2024). This low PA participation among African older adults could be because of poor policymaking, thereby creating shortcomings in the execution of laid-out PA guidelines (Balis et al. 2021). However, in a sharp contrast, a study documented high rates of vigorous PA in Ghana, with 74% reporting at least 30 min daily (Asiamah et al. 2023b). Although this did not translate into reported health benefits, it suggests that the context, type and purpose of the activity may be as important as its intensity.

These findings align with the global literature, which shows that physical activity levels decline with age and are often insufficient in older populations, particularly in low- and middle-income countries (Meredith et al. 2023).

The present study identified several recurring barriers to PA participation. Socio-demographic factors, including advanced age, female sex and low socioeconomic status, were consistently associated with lower participation levels in physical activity, as reported in previous studies (Jacob et al. 2023; Shiferaw et al. 2024; Vancampfort et al. 2018). The aforementioned demographic factors are relevant because they influence the distribution of healthcare given to this population. Low participation in PA among older adults with advanced age and low socioeconomic status increases their burden of diseases, leading to a rise in demand for geriatric care in a population plagued by scarcity (Aro, Agbo & Omole 2018; Balis et al. 2019; Taylor, Nyakotey & Kwarteng 2023).

Chronic illnesses, including diabetes, chronic pain and respiratory conditions, were also found in this study to reduce participation (Banson et al. 2023). This was consistent with other authors who noted that although PA is beneficial in this population, having one or more chronic conditions presented older adults with increased functional disability (Asiamah et al. 2023a; Awuviry-Newton et al. 2023; Vancampfort et al. 2017).

Interestingly, another study observed that only obesity was independently significantly associated with low PA levels (Tomaz et al. 2020). Having one or more comorbid conditions likely reduces one’s desire to engage in PA, thereby increasing disability. A study further highlighted gender and urban–rural disparities, with higher inactivity among women and urban residents in Nigeria (Adeloye et al. 2022). In addition to the already mentioned barriers, depression and inactivity were also found to be barriers to PA participation (Shiferaw et al. 2024), echoing global evidence of a bidirectional link between physical inactivity and psychological disorders (Schuch et al. 2019). These findings show the importance of adopting integrated approaches that address both physical and mental health needs among older adults.

Facilitators were less consistently reported but pointed to the importance of social structures and community support. A study found that strong family ties and social networks encouraged activity, while another study observed that encouragement from peers or health professionals motivated participation (Chikafu & Chimbari 2020). These findings are consistent with global evidence on the role of social cohesion in sustaining PA (Park et al. 2025). Similarly, other facilitators for PA hinged on the positive notion one had of their physical activity, as well as the advice of significant others, such as friends or healthcare providers and group-derived activities (Balis et al. 2019). Leveraging family, community and cultural institutions could therefore provide effective entry points for promoting PA in African settings.

The reviewed studies revealed significant health consequences of a lack of adequate PA. Low PA was associated with cognitive decline, stroke, chronic pain and sarcopenia (Jacob et al. 2023; Vancampfort et al. 2018). Little to no engagement in PA by older adults makes them vulnerable to mild cognitive impairments, as well as other non-communicable diseases. However, the absence of improved self-reported health outcomes despite high vigorous PA in some contexts suggests that health outcomes may depend not only on the quantity of activity but also on its type and cultural framing (Asiamah et al. 2023b).

Hence, the findings of this study highlight the need for gender-sensitive, culturally relevant and community-based interventions to promote PA participation among older adults in Africa. Policies should integrate PA promotion into chronic disease management and urban planning, while interventions should leverage social networks to encourage participation (Adeloye et al. 2022, Banson et al. 2023). Addressing depression and other mental health concerns is equally critical. Tailored strategies that consider both structural determinants (e.g. poverty, education, and employment) and individual factors (e.g. age, health conditions) are crucial for achieving sustainable improvements.

### Limitations of the study

The evidence remains limited by methodological diversity, small sample sizes and reliance on cross-sectional designs, restricting causal inference in the included studies. Most studies used self-reported PA measures, which may introduce recall and social desirability bias. Furthermore, country-specific data remains scarce, limiting opportunities for cross-country comparisons. Longitudinal and intervention studies are urgently needed to strengthen the evidence base.

### Practical implications

The findings from this study highlight the need for a culturally structured programme to promote and enhance participation and compliance with PA programmes. Older adults, irrespective of socioeconomic background, will be more likely to partake in PA if provided with exercise-friendly programmes. Activities should be practical and individualised, where practicable, to the health needs of older adults to promote compliance.

## Conclusion

This review indicates that physical inactivity is highly prevalent among older adults in Africa, with participation influenced by biomedical, social and cultural determinants. While social networks and community support promote engagement, barriers such as chronic illness, age, gender and low socioeconomic status persist. To support healthy ageing, interventions must be holistic, gender responsive, and context -specific, addressing both individual and structural determinants of health and well-being.
